# Editorial: Awareness, Treatment, and Control of Hypertension or Diabetes in India: The Impact of Public Health Promotion

**DOI:** 10.3389/fpubh.2022.906862

**Published:** 2022-05-02

**Authors:** Kavumpurathu Raman Thankappan, Meena Daivadanam, G. K. Mini, Rohina Joshi, Thirunavukkarasu Sathish

**Affiliations:** ^1^Department of Public Health and Community Medicine, Central University Kerala, Kasargod, India; ^2^Department of Women's and Children's Health, Uppsala University and Department of Global Public Health, Karolinska Institutet, Solna, Sweden; ^3^Global Institute of Public Health, Ananthapuri Hospitals and Research Institute, Thiruvananthapuram, India; ^4^Global Health, School of Population Health, University of New South Wales, Sydney, NSW, Australia; ^5^Department of Global Health, Population Health Research Institute, McMaster University, Hamilton, ON, Canada

**Keywords:** awareness, treatment, control, hypertension, diabetes, health promotion, India

In India, recent estimates show that there are 207 million people with hypertension (1) and 74 million people with diabetes (2). A nationally representative study among 1.3 million Indian adults aged 18 years and above reported a hypertension prevalence of 25.3% and a diabetes prevalence of 7.5% in 2012–14 (3). Despite the high prevalence, the awareness (men:32%, women:42%), treatment (men:25%, women:35%) and control (men:11%, women:18%) rates of hypertension in the country are low (4). Similar data for diabetes are not available from a representative sample in India and one of the articles included in this special issue fills this gap.

India is a country with huge variations in health and development indicators between states (5), consequently the prevalence of hypertension and diabetes vary across states. For example, diabetes prevalence among women aged 18 years and above ranges from 2.3% in Madhya Pradesh to 16.4% in Goa and among men, the respective figures are 2.7 and 17.9% (3). Hypertension prevalence among women ranges from 13.5% in Chhattisgarh to 36.3% in Daman and Diu, whereas in men they vary from 17.1 to 43.5%, respectively (3). Similar variation in awareness, treatment and control is also reflected in a few articles included in this issue.

The low rates of awareness, treatment and control of hypertension and diabetes are likely to lead to increased vascular and renal complications in the population (6). Thus, there is an urgent need for improved detection and treatment of these conditions through task-sharing, regular supply of medications, and strategies to improve healthier diet and physical activity (7). An intervention using mobile phone based clinical decision support system in primary care in India was effective in improving hypertension and diabetes control (8). A school based educational intervention in Kerala was found to be effective in improving hypertension control rates among teachers (9). However, unless these studies in controlled settings are scaled up the control rates are likely to be poor.

In this special issue, 11 manuscripts were submitted, and of which nine articles were published; four on hypertension, three on diabetes, one study on hypertension and diabetes, and another study on doctor-patient relationship among hospitalized patients.

Bhatia et al. analyzed the nationally representative data of about 72,000 adults (aged ≥ 45 years) and reported a hypertension prevalence of 45%. The states with a higher proportion of people below poverty line had a lower performance in the diagnosis of hypertension and states with a greater availability of doctors had a better performance of treatment-seeking behavior.

Thakur and Nangia reported a hypertension prevalence of 40% in Punjab and 26% in Haryana and a diabetes prevalence of 14 and 15%, respectively. The awareness, treatment and control rates of hypertension were 48, 31, and 18%, respectively in Punjab and 33, 26, and 12% in Haryana. These rates for diabetes were 34, 28, and 14%, respectively in Punjab and 29, 22, and 14% in Haryana.

Cao et al. explored factors associated with awareness, treatment, and control of hypertension among adults in Kerala state. The authors reported that psychosocial factors, better engagement with health services in hypertension management, as well as giving more attention to body fat control and largely male-related behaviors such as alcohol consumption are likely to improve hypertension management.

Sreedevi et al. reported the need for control of hypertension among diabetes patients in Kerala state. Most of the diabetes patients in the study did not achieve the target blood pressure control. They suggested effective and stringent screening measures to control hypertension in this population.

Jebasingh and Thomas emphasized the need for physicians to be aware of low birth weight (LBW) as a potential cause for early-onset hypertension and the importance to elicit this history from the mother of the patient. The authors also suggest that LBW babies need to be provided with adequate nutrition and should not be overfed with additional calories which could result in early-onset hypertension.

Mathur et al. provided the prevalence, awareness, treatment and control of diabetes and associated factors amongst adults using a nationally representative sample of 10,659 adults in India. A prevalence of 9% and low rates of awareness (56%), treatment (36%), and control (16%) of diabetes were reported. The authors emphasized the need for continuous monitoring and surveillance of diabetes and the role of comprehensive health promotion and management interventions to achieve the World Health Organization global non-communicable disease voluntary targets by 2025.

Rahul et al. studied the level of glycaemic control among patients with diabetes using a standardized modular based training including the importance of adherence to antidiabetic medication delivered through front line health workers in a randomized controlled trial in Kerala state. The study reported promising results on improving glycaemic control at 6 months after the intervention. These findings emphasize the benefits of utilizing existing health service personnel to control diabetes.

Using a randomized controlled trial, Joshi et al. reported the findings of a pilot study on the development, testing and integration of a multidisciplinary program targeted to address the co-management of tuberculosis and diabetes in a rural primary healthcare setting in Andhra Pradesh. Even though the awareness about diabetes and tuberculosis (TB) and cardiovascular risk increased among non-physician health workers over 8 months, there was no significant variation in the mean blood glucose level in the control and intervention groups. The study findings suggest that co-management of TB and diabetes within the existing health care systems is likely to be feasible.

Gala et al. explored experiences, perceptions and expectations of doctor-patient relationship among recently hospitalized patients in Karnataka state of India. They reported a more positive doctor-patient relationship for those with primary care providers, which is important for improving hypertension and diabetes care.

In summary, the studies published in this special issue reiterate that the prevalence of hypertension and diabetes is high in India, but the rates of awareness, treatment and control are unacceptably low. These findings clearly emphasize the importance of health promotion and other evidence-based interventions to improve these rates to reduce complications related to these health conditions in India.

## Author Contributions

All authors listed have made a substantial, direct, and intellectual contribution to the work and approved it for publication.

## Conflict of Interest

The authors declare that the research was conducted in the absence of any commercial or financial relationships that could be construed as a potential conflict of interest.

## Publisher's Note

All claims expressed in this article are solely those of the authors and do not necessarily represent those of their affiliated organizations, or those of the publisher, the editors and the reviewers. Any product that may be evaluated in this article, or claim that may be made by its manufacturer, is not guaranteed or endorsed by the publisher.
